# An Optically Transparent Metasurface-Based Resonant Cavity Fed by Patch Antenna for Improved Gain

**DOI:** 10.3390/ma12233805

**Published:** 2019-11-20

**Authors:** Qinlong Li, Xiaoming Chen, Xin Hu

**Affiliations:** 1 School of Information and Communications Engineering, Xi’an Jiaotong University, Xi’an 710049, China; liql519@mail.xjtu.edu.cn; 2 School of Electronic Engineering, Beijing University of Posts and Telecommunications, Beijing 100876, China

**Keywords:** metasurface, transparent conductive film, patch antenna, gain improvement

## Abstract

An optically transparent metasurface (MS) is proposed to design a resonant cavity fed by a patch antenna operating at 5.6 GHz. In the proposed MS, a transparent micro metal mesh conductive (MMMC) film is used as the transparent conducting film (TCF), and it has a high optical transmittance of more than 75% and a low sheet resistance of 0.7 Ω/sq. The MS is composed of a layer of glass substrate and a layer of MMMC film. The unit cell of MS consists of a square patch using MMMC film patterned on a square glass substrate. The transparent MS, patch antenna, ground plane, and air-filled half-wavelength cavity form a resonant cavity antenna, to achieve an improved gain. The MS is designed, optimized and analyzed using the EM simulation software CST. Results show that the MS can improve the simulated boresight gain from 4.7 to 13.2 dBi by 8.5 dB, without affecting the impedance bandwidth (IMBW) much. The losses of MS with different values of sheet resistance are also studied, showing the MS using MMMC with sheet resistance of 0.7 Ω/sq has very small losses.

## 1. Introduction

In recent years there has been increased interest in thin-film, electromagnetically sensitive surfaces known as metasurfaces (MS). Metasurfaces are typically defined as a designed structures with lattice constants and thicknesses bellow the Bragg limit. Various kinds of MSs have been reported to improve the performances of antennas [1,2,3,4,5,6,7,8]. For example, due to the 0° reflection phase of an artificial magnetic surface (AMC), the antenna can have a higher boresight gain with a lower profile by using AMC as ground plane below the radiating element, compared with perfect electrical conductor ground [1]. To enhance the gain of antenna, gradient-refractive index (GRIN) MS can be used for phase compensation to transform the quasi-sphere Electromagnetic (EM) wave radiated by an antenna to a near planar wave [2,3]. In reference [4], with a MS placed above a crossed slot circular polarized antenna, the bandwidth and gain could be increased. By using a MS above a horn antenna, the polarization can be rotated 90° [5,6]. In reference [7], the MS is used to change the linearly polarized EM waves from an antenna into circularly polarized EM waves. In reference [8], a radar cross-section (RCS) reduction technique by using the coding diffusion MS is presented, and the MS is optimized through a random optimization algorithm.

Recently, some microwave components, such as antennas and couplers, have been designed using transparent conductive film (TCF) for the purpose of aesthetics [9,10,11,12,13]. There are a lot of kinds of optically transparent and electrically TCFs manufactured by Cima Nano Tech company using self-assembling nanoparticle technology [9,10,11]. The sheet resistance of micro metal mesh conductive (MMMC) film is much lower than other TCFs such as indium tin oxide (ITO) [12], AgHT-8 and AgHT-4 [13]. Therefore, the microwave components using MMMC film will have less Ohmic loss, and this conductive film has already been applied in antenna [9], branch-line coupler [10], phase shifter [11].

In this paper, we propose an optically transparent MS using MMMC film to design a resonant cavity fed [14,15,16,17] by a patch antenna operating at 5.6 GHz. To the best knowledge of the authors, no one has used MMMC film to design a MS for the resonant cavity antenna [14,15,16,17]. The unit cell of MS consists of a square patch using MMMC film patterned on a square glass substrate. Two models, including a superstrate reflection model (SRM) and defect cavity model (DCM), are used to analyze the MS and the cavity, respectively. The proposed MS has a sufficiently strong reflectivity of more than 0.8 using SRM. However, the whole resonant cavity including the MS, ground plane, and air-filled half-wavelength cavity, has a passband at the resonant frequency of 5.6 GHz using DCM. The losses of MS with different values of sheet resistance are also studied, indicating the MS using the proposed MMMC film also has very small losses. A patch antenna is used for studying the gain enhancement of the MS. Measurement results demonstrate that the proposed MS has little influence on the impedance bandwidth (IMBW) of the patch antenna, whereas it has the ability to improve the measured boresight gain by 7 dB (from 5.2 to 12.2 dBi) at the resonant frequency of 5.6 GHz.

## 2. Antenna Design

**Figure 1:**
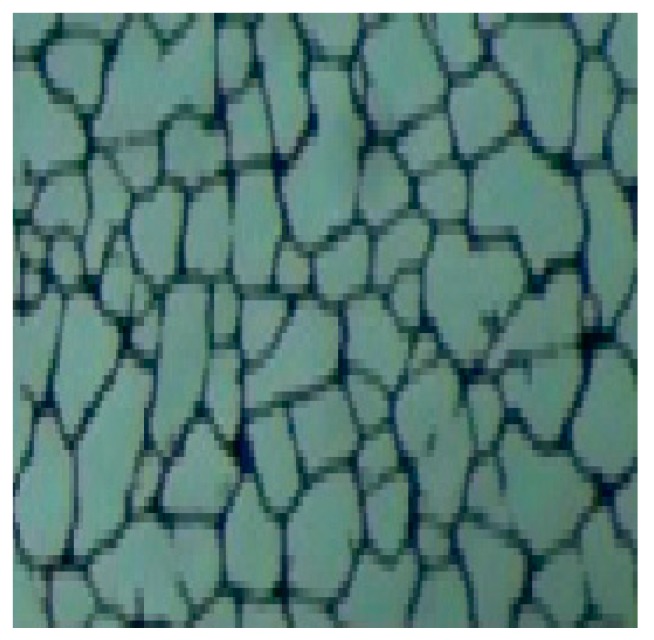
MMMC film viewed through microscope.

In our design, a transparent micro metal mesh conductive (MMMC) film is used as the transparent conducting film (TCF), which is constructed by Cima Nano Tech using SANTE self-assembling nanoparticle technology. It has a high optical transmittance of more than 75% and a low sheet resistance of 0.7 Ω/sq. The MMMC film consists of two layers, a conductive layer made of silver laminated over a layer of polyethylene terephthalate (PET). Figure 1 shows the transparent MMMC film viewed through microscope. It can be seen that conductive strips are distributed on the PET layer randomly. The optical transparency of the mesh material is quantitatively calculated by the percentage of non-metal strips area to the total area of the material, which is given as Equation (1). With the increase of non-metal strips area, the transparency will increase:

(1) $$\mathrm{Optical}\;\mathrm{transparency}=\frac{A_{Non-metal}}{A_{total}}\cdot 100\%$$

**Figure 2:**
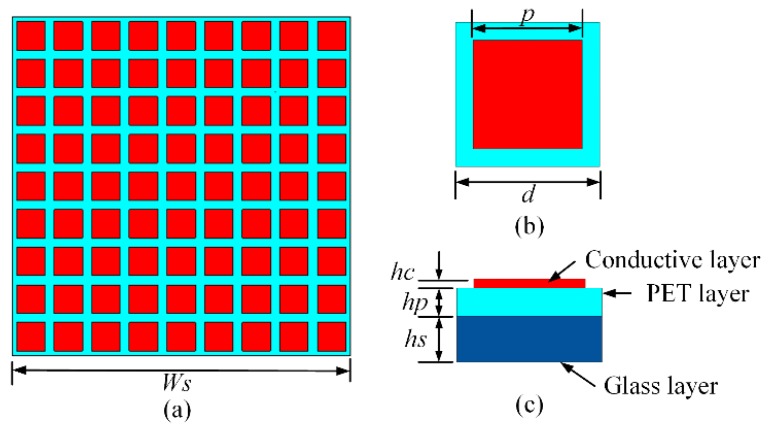
Proposed MS: (**a**) top view of MS, (**b**) top view of unit cell, and (**c**) side view of unit cell.

**Figure 3:**
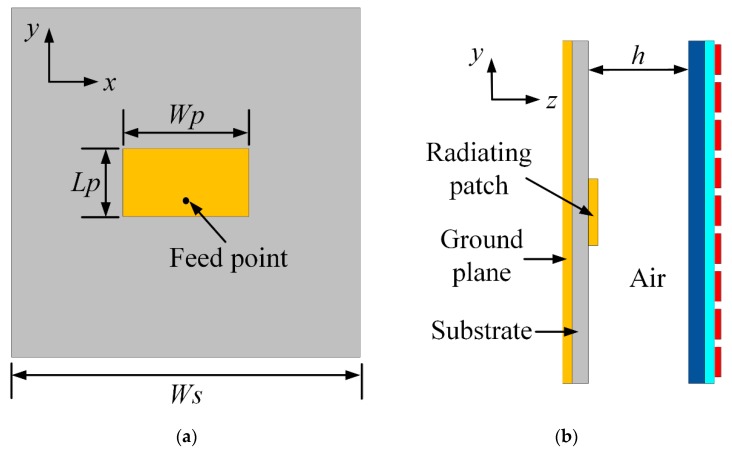
(**a**) The geometry of patch antenna (**b**) MS antenna.

**Figure 4:**
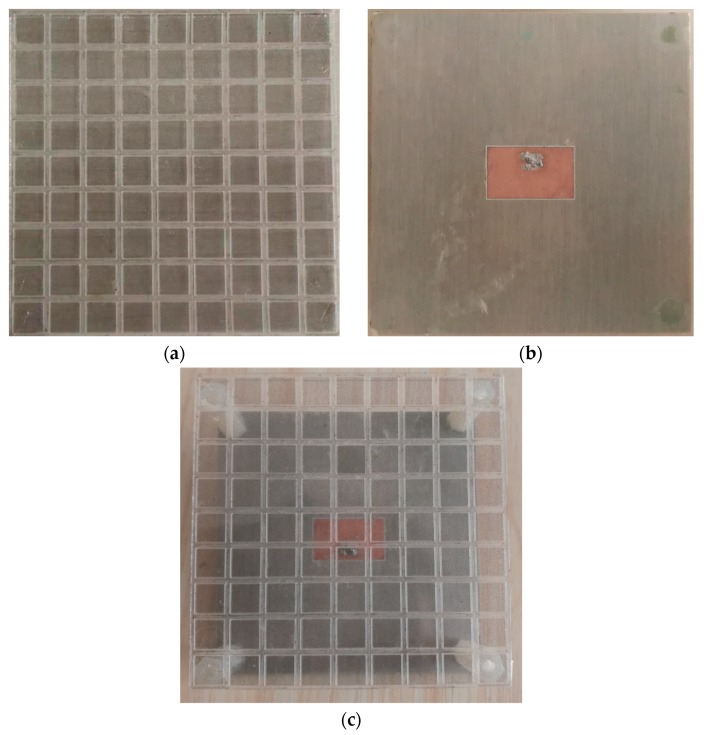
Prototype of (**a**) MS (**b**) patch antenna (**c**) MS antenna.

The configuration of designed MS is shown in Figure 2, where 81 unit cells are placed periodically in a 9 × 9 layout as shown in Figure 2a, with a total size of *Ws* × *Ws* = 81 × 81 mm^2^. Each unit cell is a square patch using MMMC film patterned on a square glass substrate with a relative permittivity of 5.7, as shown in Figure 2b. The PET has a relative permittivity of 5.1. To verify the gain improvement of the designed MS, a patch antenna shown in Figure 3a is proposed. It has a planar structure, comprising a square substrate, a rectangular radiating patch at the top side of substrate and a square metallic ground at the bottom side. The total dimension of the patch antenna is Ws × Ws = 81 × 81 mm^2^. The substrate is made of Rogers 4350B, with a thickness of 0.8 mm, a relative permittivity of 3.5 and a loss tangent of 0.004. To feed the antenna, a coaxial cable is used with inner pin connected to the radiating patch via a hole through the Rogers substrate, while the metal outside of coaxial cable is soldered on the metallic ground. As shown in Figure 3b, the MS is above the patch antenna for gain improvement at a height of *h*, forming MS antenna. Parameters of the MS antenna are listed in Table 1 and used for fabrication of the prototype. In the fabrication process of MS, we used the LPKF prototype machine to cut lines on the MMMC film, and then used a knife to scrape the unnecessary parts. In the prototype, four nylon posts at four corners are deployed to support the height between the patch antenna and the MS for measurement. The fabricated MS, patch antenna, and MS antenna are shown in Figure 4.

## 3. MS Study

### 3.1. Operation Principle

In the proposed design, the transparent MS has a characteristic of partial but high reflection, acting as a partially reflective surface (PRS). Thus, the MS antenna in Figure 3b including the patch antenna, MS, ground plane, and air-filled half-wavelength cavity, can be regarded as a resonant cavity antenna [14,15,16,17]. In this antenna, the EM waves emitted from patch antenna undergo multiple reflections and transmissions within the air-filled cavity between the metallic ground and MS. When all the waves transmitted through the PRS are in phase [18], the antenna will have the maximum gain in boresight direction. An antenna’s directivity can be enhanced significantly using the multiple reflections between the ground plane and the PRS. The operating frequency of the resonant cavity antenna can be expressed on the following equation [18]:

(2) $$f=\frac{c}{4\pi h}(\varphi_{prs}+\pi -2n\pi ),n=0,1,2\ldots$$

where *h* is the cavity height, *φ_prs_* is the reflection phase of the PRS and *π* is the reflection phase of the ground plane.

**Figure 5:**
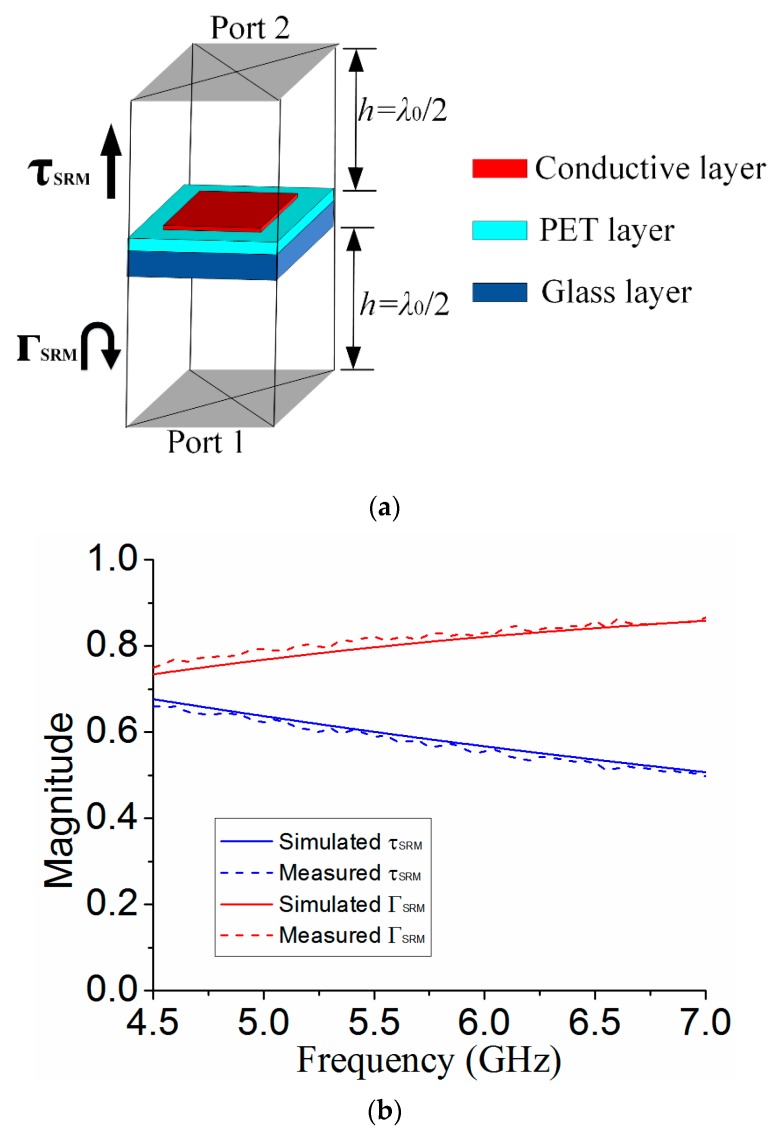
(**a**) Unit cell of the MS using SRM (**b**) simulated and measured reflection and transmission magnitude.

The proposed MS can be analyzed using superstrate reflection model (SRM) and defect cavity model (DCM) as follows [19,20]. In order to obtain high gain of antenna, the MS should have sufficiently strong reflectivity (0.6–0.9) [21]. The reflection magnitude of the proposed MS can be calculated using SRM depicted in Figure 5a, by using the template of frequency-selective surface (FSS) in CST. The unit cell in Figure 5a has the same size as in Figure 2b,c. After applying linearly polarized plane EM wave incident from port 1, the S_11_ and S_21_ between two ports can be calculated by using periodic boundary in the model. The reflection and transmission magnitude for SRM are also measured. It can be seen that both the simulated and measured reflection magnitude $\left|\Gamma_{\mathrm{SRM}}\right|$ of the proposed MS is about 0.8 at the desired resonant frequency of 5.6 GHz, as shown in Figure 5b, satisfying the requirement for PRS within 0.6–0.9.

**Figure 6:**
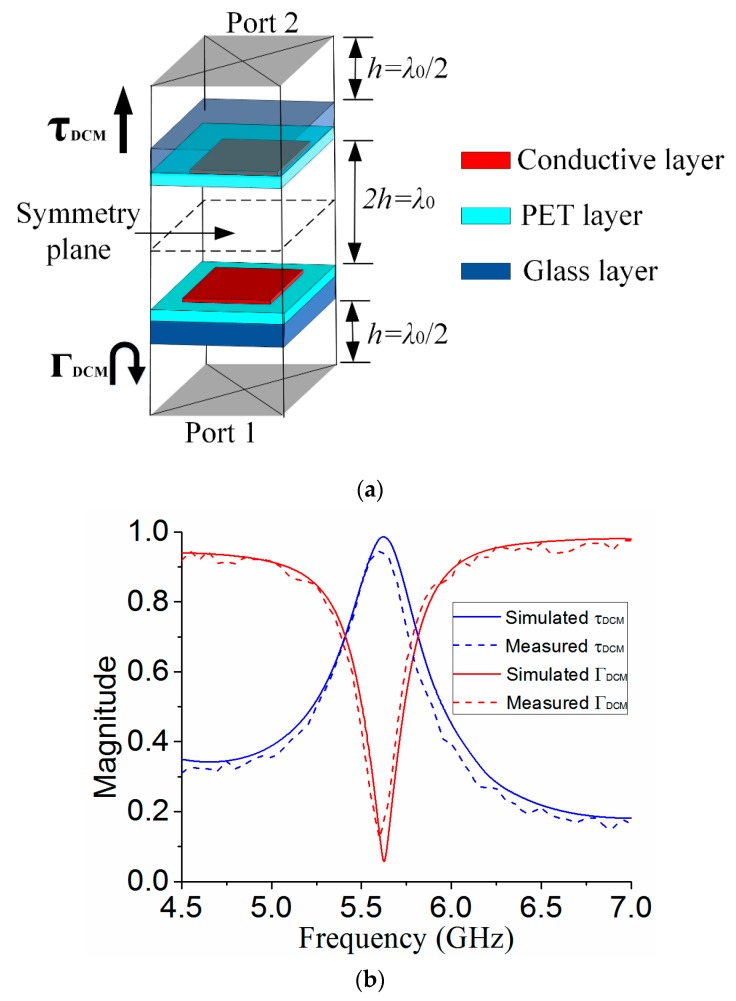
(**a**) Unit cell of the resonant cavity using DCM (**b**) simulated and measured reflection and transmission magnitude.

Different from SRM to evaluate the reflection magnitude of MS, DCM is developed to study the whole resonant cavity including the MS, ground plane, and air-filled half-wavelength cavity [22]. The equivalent unit cell of the resonant cavity can be analyzed using mirror image theory as shown in Figure 6a. The metallic ground plane generates the electric image of the MS, and the unit cell of the resonant cavity consists of the unit cell of MS and its image through the symmetry plan. As the same as the unit cell of MS, the S-parameters between ports 1 and 2 of the unit cell of the resonant cavity are also calculated using periodic boundary, and the simulation results are shown in Figure 6b. Although the MS alone has a high reflectivity, a passband is generated at the resonant frequency of 5.6 GHz for the whole resonant cavity. The transmission bandwidth with $\left|\tau_{\mathrm{DCM}}\right|\geq 0.7$ is 5.4–5.8 GHz, indicating that most of the EM power in the cavity can be transmitted through the MS in this frequency band [21]. For measurement, the measured reflection and transmission magnitude for DCM have good agreements with simulation results. With the height *h* = 29.5 mm, thickness *hp* = 0.012 mm and *hs* = 1 mm unchanged, the desired resonant frequency of the cavity is achieved by tuning the length *p* and *d* of the unit cell. In our design, *d* = 9 mm and *p* = 6.8 mm are selected.

### 3.2. Effects of Sheet Resistance

**Figure 7:**
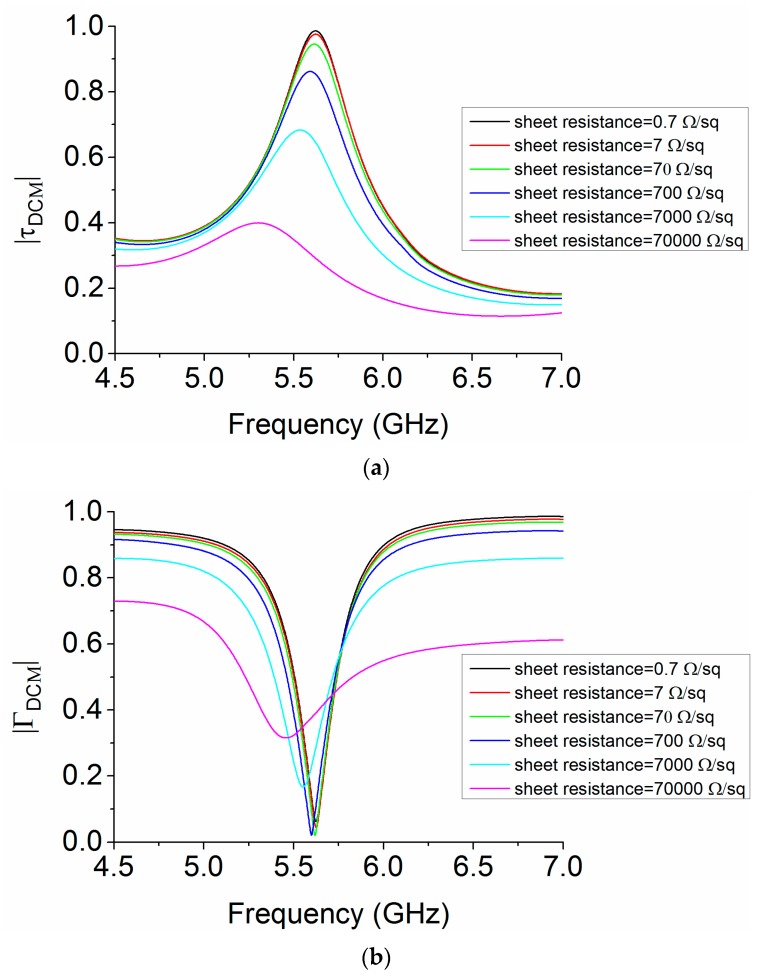
Computed reflection and transmission magnitude using different values of sheet resistance (**a**) $\left|\tau_{\mathrm{DCM}}\right|$ (**b**) $\left|\Gamma_{\mathrm{DCM}}\right|$.

**Figure 8:**
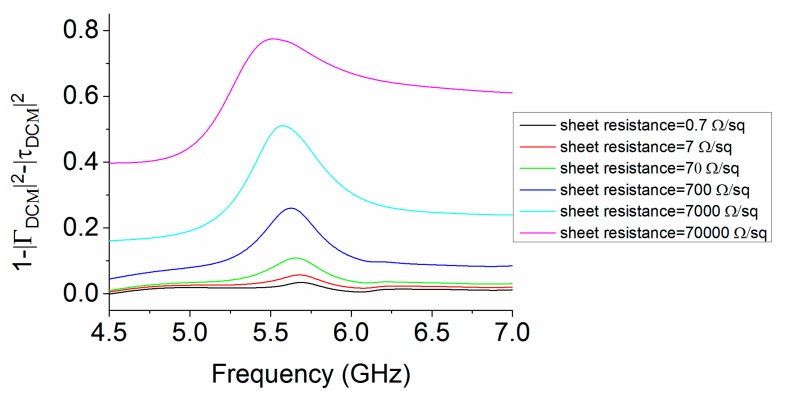
Losses of MS with different values of sheet resistance.

The TCF used for the design of MS is an MMMC film. The conductive layer of MMMC film in Figure 1 has a sheet resistance of 0.7 Ω/sq, and a thickness of 0.005 mm. It is essential to study the losses of MS by using the MMMC film. A parametric study has been carried out using simulation to study the effect of sheet resistance on the losses of MS. The DCM is used to analyze the losses. With the different values of sheet resistance, and a thickness remaining unchanged at 0.005 mm, the reflection magnitude $\left|\Gamma_{\mathrm{DCM}}\right|$ and transmission magnitude $\left|\tau_{\mathrm{DCM}}\right|$ can be computed by simulation in CST, and the results are shown in Figure 7. The losses of MS in the cavity can be calculated using Equation (3). Thus, using the results in Figure 7, the losses of MS with different values of sheet resistance in the cavity are calculated as shown in Figure 8. It can be seen that, with the increase of sheet resistance, the losses become larger. In our design, the conductive layer of MMMC film has a sheet resistance of 0.7 Ω/sq, and the losses are less than 0.03, as shown in Figure 8, which are very small. The results with sheet resistance less than 0.7 Ω/sq are nearly unchanged compared with the results with sheet resistance of 0.7 Ω/sq, so they are not shown for brevity.

(3) $$\mathrm{Losses}=1-{\left|\tau_{\mathrm{DCM}}\right|}^{2}-{\left|\Gamma_{\mathrm{DCM}}\right|}^{2}$$

### 3.3. Comparison with Copper

**Figure 9:**
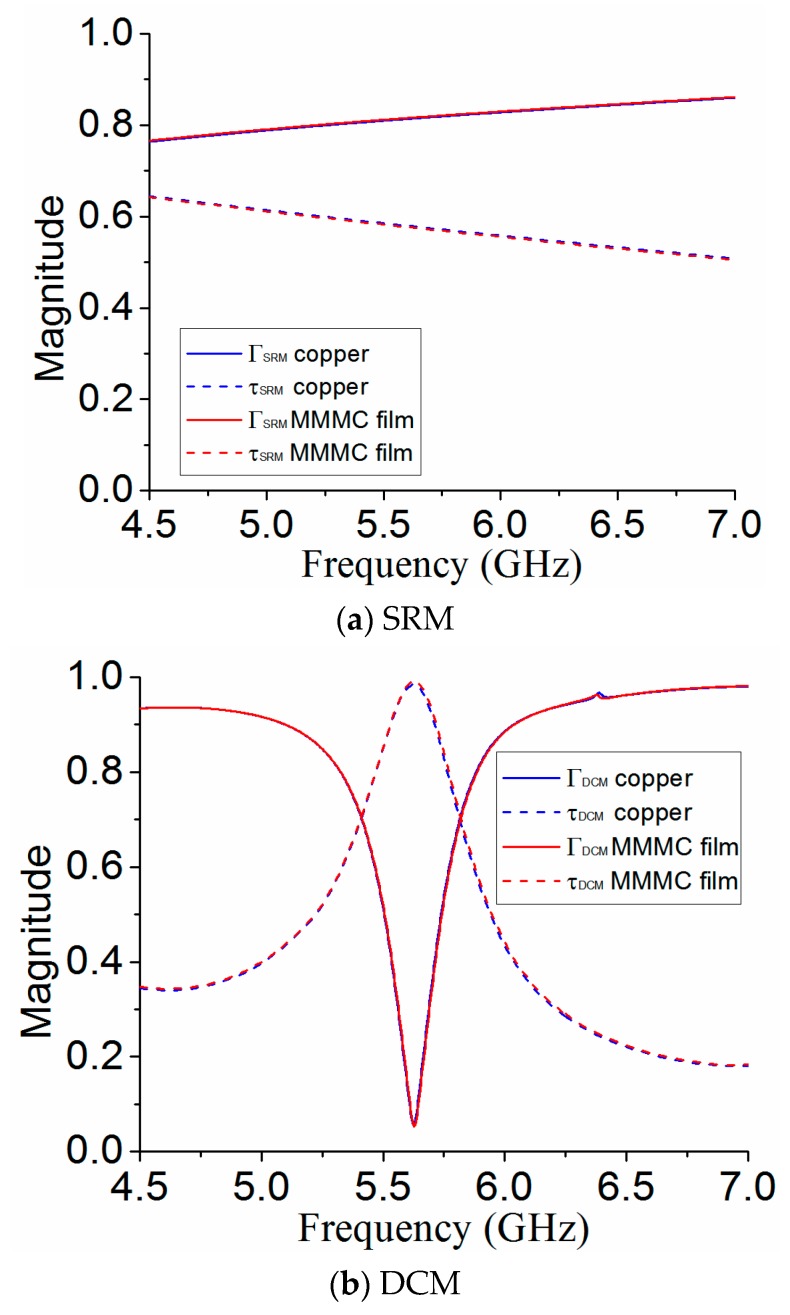
Performances comparison of MS between using MMMC film and copper.

The conductivity of the MMMC is high, but it is still orders of magnitude worse than a material like copper. Therefore, it is significant to compare the results of MS if the transparent conductive layer in the MS is replaced by copper. The copper’s conductivity in the simulation is 5.8 × 10^7^ S/m, with the same thickness of 0.05 mm, so the equivalent sheet resistance can be calculated as 0.0034 Ω/sq. The results are shown in Figure 9. It can be seen that there is little difference for the performances of MS for both SRM and DCM when the MMMC film is replaced by copper.

## 4. Simulation and Measurement Results

**Figure 10:**
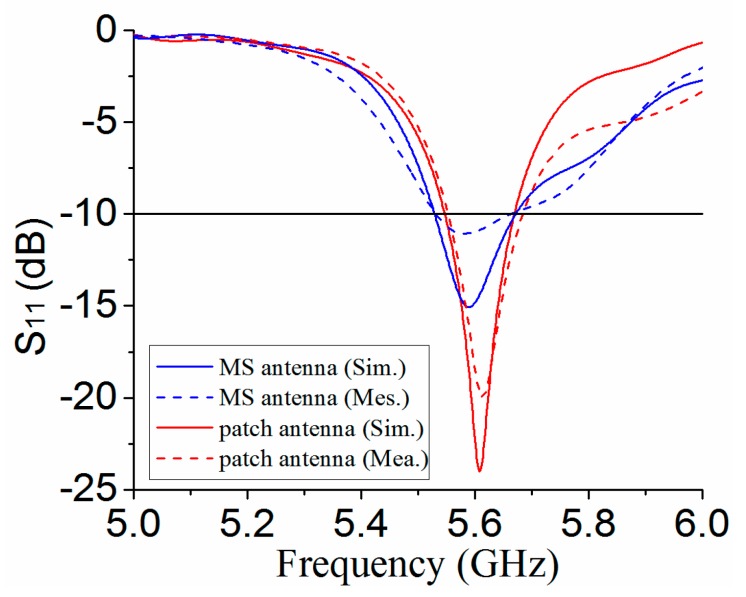
Simulated and measured S_11_ for patch antenna and MS antenna.

The full-wave simulation tool CST is used to carry out simulations for optimization and analysis of both patch and MS antenna in Figure 3a,b. For verification, the fabricated antennas are measured by a vector-network analyzer (VNA) for S_11_ and the antenna measurement system Satimo Starlab for boresight gains and radiation patterns. The simulation and measurement results for both patch antenna and MS antenna have good agreement. Figure 10 shows the simulated and measured S_11_ for both MS antenna and patch antenna. The simulated impedance bandwidths (IMBWs) with S_11_ < −10 dB for the MS antenna and patch antenna are 5.53–5.67 GHz and 5.55–5.67, respectively. The measured IMBWs are 5.52–5.66 GHz and 5.56–5.69 GHz for both the MS antenna and patch antenna. Therefore, the use of MS for antenna gain improvement has little influence on the IMBW of the patch antenna.

**Figure 11:**
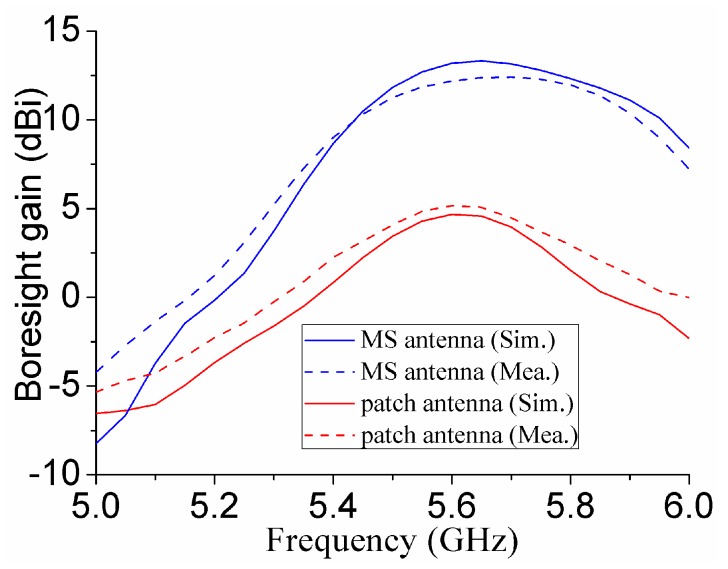
Simulated and measured boresight gains for patch antenna and MS antenna.

**Figure 12:**
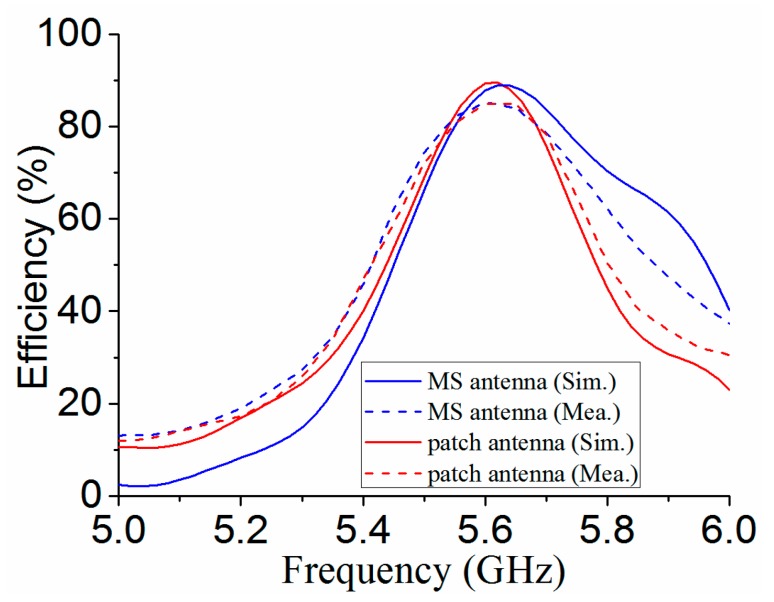
Simulated and measured efficiencies for patch antenna and MS antenna.

Figure 11 shows the simulated and measured boresight gains for the MS antenna and patch antenna. It can be seen that the MS can improve the boresight gains from 4.7 dBi to 13.2 dBi for simulation, while it can enhance boresight gains from 5.2 to 12.2 dBi for measurement at the resonant frequency of 5.6 GHz. Figure 12 shows the simulated and measured boresight efficiencies for the MS antenna and patch antenna. The results show that simulated efficiencies of both patch antenna and MS antenna are more than 85% at 5.6 GHz, while the measured efficiencies are more than 80% at 5.6 GHz. These results also indicate the use of MS has very small losses.

**Figure 13:**
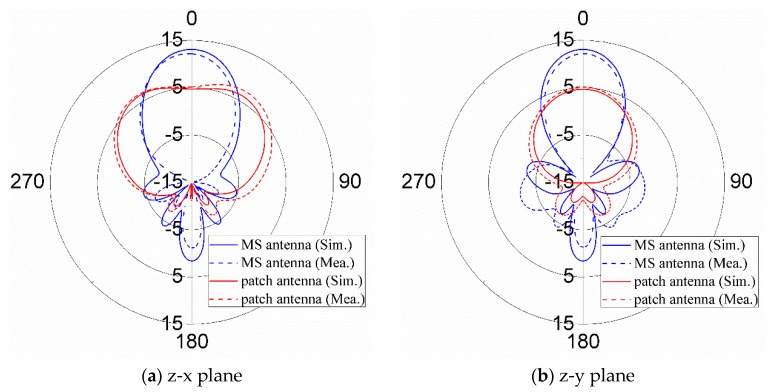
Simulated and measured radiation patterns.

Figure 13 shows the simulated and measured radiation patterns of the MS antenna and patch antenna at 5.6 GHz in both z-x and z-y planes. In Figure 13a, it can be found that the patch antenna has half-power beam widths (HPBWs) of 68° and 69.8° in the z-x plane for both simulation and measurement, respectively. The MS antenna has much narrower HPBWs of 32° and 33° in z-x plane for both simulation and measurement. Similarly, the MS can also reduce the HPBWs significantly in z-y plane. It can be seen in Figure 13b that the patch antenna has the simulated and measured half-power beam widths (HPBWs) of 116.4° and 122.7° in z-y plane, respectively. However, the simulated and measured HPBWs of MS antenna are 35.4° and 36.5°, much less than the corresponding results of patch antenna in z-y plane. These results indicate that, with the use of MS, the HPBWs can be reduced significantly, hence improving the boresight gains of patch antenna.

## 5. Conclusions

An optically transparent MS using MMMC film is proposed to design a resonant cavity fed by a patch antenna. The TCF used in the proposed MS is MMMC film, with a low sheet resistance of 0.7 Ω/sq and a highly optically transmittance more than 75%. The unit cell of MS consists of a square patch using MMMC film patterned on a square glass substrate. Two models, including SRM and DCM, are used to analyze the operation principle of the MS. The losses of MS with different values of sheet resistance are also studied, showing the MS using MMMC with sheet resistance of 0.7 Ω/sq has very small losses. Measurement results show that the proposed MS antenna and patch antenna have similar IMBWs, but the MS antenna has a much higher gain compared with the patch antenna.

## Figures and Tables

**Table 1:** Dimensions of the proposed antenna (unit: mm).

*W_s_*	*W_p_*	*L_p_*	*p*	*d*	*h*	*hc*	*hp*	*hs*
81	22	13	9	6.8	29.5	0.005	0.012	1
